# Pharmacokinetic and Toxicological Evaluation of a Zinc Gluconate-Based Chemical Sterilant Using In Vitro and In Silico Approaches

**DOI:** 10.1155/2017/5746768

**Published:** 2017-01-19

**Authors:** Carlos F. Araujo-Lima, Rafael J. M. Nunes, Raphael M. Carpes, Claudia A. F. Aiub, Israel Felzenszwalb

**Affiliations:** ^1^Department of Biophysics and Biometry, Rio de Janeiro State University, Boulevard 28 de Setembro, 87 Fundos, 4° Andar, 20551-030 Rio de Janeiro, RJ, Brazil; ^2^Department of Genetics and Molecular Biology, Federal University of the State of Rio de Janeiro, Rua Frei Caneca, 94 Centro, 20211-040 Rio de Janeiro, RJ, Brazil

## Abstract

Sclerosing agents as zinc gluconate-based chemical sterilants (Infertile®) are used for chemical castration. This solution is injected into the animal testis, but there are not enough evidences of its safety profiles for the receivers. The present work aimed to establish the pharmacokinetics and toxicological activity of Infertile, using in vitro and in silico approaches. The evaluation at the endpoint showed effects in a dose-dependent manner. Since necrosis is potentially carcinogenic, the possible cell death mechanism could be apoptosis. Our data suggested that Infertile at 60 mM presented risk for animal health. Even though Infertile is a licensed product by the Brazilian Ministry of Agriculture, Livestock and Supply, it presented a high mutagenic potential. We suggest that the optimal dose must be less than 6 mM, once, at this concentration, no mutagenicity or genotoxicity was observed.

## 1. Introduction

The absence of canine birth control for domestic or stray dogs in big cities represents a public health and animal welfare problem [1]. When abandoned, these animals are at risk of being attacked by humans or other animals or can even become reservoirs of zoonotic diseases [2, 3]. In order to control the canine population, numerous strategies have been described and used to prevent/stop the reproductive cycle, including surgery, hormonal modulation, and chemical and immunological methods. Surgical intervention is a guaranteed but expensive method, as it requires a hospital setting and involves risks associated with anesthesia and surgical wound infection [4].

Many dog owners argue that this method is invasive and incompatible with animal welfare [5]. As a tactical control method, hormonal steroids (such as estrogen-progesterone or progesterone only) have been orally administered to laboratory dogs to suppress ovulation [6, 7]. However, there are side effects, such as pyometra, an inflammatory reaction in the uterus, followed by bacterial infection, and cystic endometrial hyperplasia [8].

Chemical castration is performed by injecting a sclerosing agent into the animal's testis, epididymis, or* vas deferens*. This procedure is irreversible due to its action on germ cells, but no side effects have been reported [9]. Sclerosing agents act via systemic immune response, causing the rupture of the testis barrier and death of Sertoli cells. These agents can also induce local inflammation and the release of testis antigens [10]. Solutions are injected into the animal's testis, resulting in testicular germ cell atrophy, impairment of spermatogenesis, and fibrous occlusion of the* vas deferens* or the epididymis [11].

**Figure 1:**
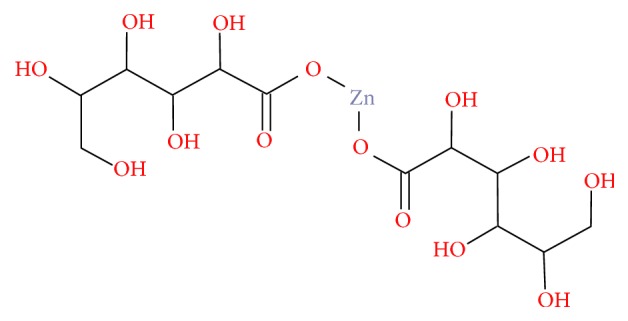
Zinc gluconate chemical structure.

Zinc is an important mineral for spermatogenesis and semen constitution. Nevertheless, in very high concentrations, zinc acts as an inhibitor of germ cell division and replication and leads to nucleus and cell membrane fragmentation [12], as reported in other studies that show zinc toxicity during animal development and breeding [13]. Chemical sterilants have been on the market since the year 2000, when Neutersol® was approved by the Food and Drug Administration (FDA) [14, 15]. Still, they have been tested in dogs since the 1970s [16–18] and other animals since the 1950s [19]. Recently, several chemical castration agents have been approved by the FDA and other health and sanitation agencies around the world, including zinc gluconate-based products (Figure 1), such as Testoblock® (BioRelease Tech., Birmingham, AL, USA) [6] and Infertile (RhobiPharma Ind. Farm., Hortolândia, SP, BR). A study demonstrated that Infertile is an effective sterilant for it induces changes in testis germ cells, producing fewer sperm cells and high rates of morphological defects [20].

All products used for human and animal health should be evaluated for their potential to induce DNA damage. The recommendation is to proceed with at least two in vitro genotoxicity tests before performing animal testing. In general, the first test to assess the toxicity of chemical compounds is the* Salmonella*/Microsome test, or Ames test, which shows patterns of mutation in DNA structure. Even if no positive results are observed for the Ames test, it is necessary to evaluate clastogenicity and chromosomal aberrations (damage to coiled nuclear DNA) in eukaryotic cells using micronucleus test [21].

The license from Brazilian Ministry of Agriculture, Livestock and Supply (MAPA) (9427/2008) does not provide enough data on the mutagenicity and carcinogenicity of Infertile. As specified in the ordinance MAPA 74/1996, the agency determines that possible mutagenic, carcinogenic, and teratogenic effects must be declared by the manufacturers of veterinary pharmaceuticals. According to Brazilian animal health law, there is no need to evaluate these endpoints to obtain the license. Since 2006, though, Brazil is a cosignatory of OECD, so all products used for human and animal health have to be tested for their mutagenic, genotoxic, carcinogenic, and teratogenic potential. Chemical sterilization has been more economically and practically feasible. However, little is known about its mutagenic and genotoxic potential. The present work aims to investigate the pharmacokinetic and toxicological potential of zinc gluconate, using in silico and in vitro methods.

## 2. Materials and Methods

### 2.1. In Silico Approach

We used a modular toxicological predictive QSAR framework algorithm (LAZAR in silico toxicology, https://lazar.in-silico.ch/predict) [22] based on similarity of chemical alerts. To perform the toxicological prediction in LAZAR, we designed zinc gluconate chemical structure using ChemDraw and obtained the SMILE string. With the SMILE string, we predicted absorption, distribution, metabolism, excretion, and toxicological parameters (ADMET) based on QSAR similarity, using the pharmacokinetic algorithm pkCSM (http://bleoberis.bioc.cam.ac.uk/pkcsm/prediction) [23].

### 2.2. In Vitro Biological Approach

#### 2.2.1. Test Compound

Toxicological tests were performed with ampoules of Infertile, lot 001/09, kindly provided by Dr. Helena de Godoy Bergallo, Laboratory of Small Mammal Ecology, Rio de Janeiro State University (UERJ). Dimethyl-sulfoxide (DMSO) at 10% was used to dilute the compound for the tests. The presence of many hydroxyl radicals in zinc gluconate structure may contribute to biological activity and solubility of the compound.

#### 2.2.2. Salmonella/Microsome Mutagenicity Test

The features of* Salmonella enterica *serovar Typhimurium standard strains TA97, TA98, TA100, TA102, and TA104 from the authors' stock were used as described by Cardoso et al. [24] in the mutagenicity assay.

The test tube contained a mixture of 100 *μ*L of one of the Infertile concentrations (6, 12, 30, and 60 mM) plus either 500 *μ*L sodium-phosphate buffer (27.6 g/L NaH_2_PO_4_·H_2_O and 28.4 g/L Na_2_HPO_4_; 0.2 M, pH 7.4) or a metabolic fraction (S9 mix 4%; Molecular Toxicology Inc., MoltoxTM, USA) composed of a homogenate of Sprague-Dawley rat liver cells pretreated with polychlorinated biphenyl (Aroclor 1254), as well as 100 *μ*L of the bacterial suspension (2 × 10^9^ cells/mL). After 20 minutes of preincubation at 37°C, 2 mL of top agar (7 g/L agar; 5 g/L NaCl; 0.0105 g/L L-histidine; 0.0122 g/L biotin; pH 7.4, 45°C) were added to the test tube, and the final mixture was poured onto a Petri dish with minimal agar (15 g/L agar, Vogel-Bonner E medium 10x (10 g/L MgSO_4_·7H_2_O; 100 g/L C_6_H_8_O_7_·H_2_O; 500 g/L K_2_HPO_4_; 175 g/L Na(NH_4_)HPO_4_·4H_2_O) containing 20 g/L glucose. This final mixture was incubated at 37°C for 72 h, and the* His*^*+ *^revertant colonies were counted. The positive controls for assays in the absence of S9 mix were 4-Nitroquinoline-N-oxide (4-NQNO) (CAS: 56-57-5) at 1.0 *μ*g/plate, for TA97 and TA98; sodium azide (SA) (CAS: 26628-22-8) at 0.5 *μ*g/plate, for TA100; Mitomycin C (MM C) (CAS: 50-07-7) at 0.5 *μ*g/plate, for TA102; Methyl-methane sulfonate (MMS) at 50 *μ*g/plate (CAS: 66-27-3) for TA104. In the presence of S9 mix, the positive controls were 2-Aminoanthracene (2-AA) (CAS: 613-13-8) at 1.0 *μ*g/plate for TA97 and TA100; and Benzo[a]Pyrene (B[a]P) (CAS: 50-32-8) at 20 *μ*g/plate for TA98, TA102, and TA104. All the chemicals were purchased from Sigma Co. (St. Louis, USA). The substance or sample was considered positive for mutagenicity when the number of revertant colonies in the assay was at least twice the number of spontaneous revertants (mutagenicity index, MI ≥ 2) and when a significant response to the analysis of variance (ANOVA, *P* ≤ 0.05) and reproducible positive dose-response curve (*P* ≤ 0.01) were found. MI was calculated by dividing the number of* His*^*+*^ induced in the sample by the number of* His*^*+*^ in the negative control. All the experiments were done in triplicate and repeated at least twice [25, 26].

#### 2.2.3. Survival Experiments

Quantitative evaluations were made to determine the cytotoxic effects for all the drug concentrations. In this step, 10 *μ*L of the treated bacterial suspension was diluted in a saline solution (NaCl 9 g/L-0.9%). Then, 100 *μ*L of the solution was put on a Petri dish with Luria Bertani (LB) agar and incubated at 37°C for 24 h. The total dilution was 10^−7^ fold. Colonies were counted and a survival percentage was calculated in relation to the negative control. The compound was considered cytotoxic when its survival rate was lower than 70% of bacterial survival, a significant response to one*-*way analysis of variance (ANOVA, *P* ≤ 0.05) and reproducible dose-response curve (*P* ≤ 0.01) [24].

#### 2.2.4. Micronuclei in Cell Culture

The RAW264.7 macrophages were cultured in circular coverslips at 24-well plates with 950 *μ*L essential Minimum Eagle Medium (MEM) Ca^++^, 1.8 mM, pH 7.6 (Gibco), supplemented with 1.76 g/L NaHCO_3_, 0.88 g/L pyruvate, 21.6 mg/L aspartic acid, and 16.8 mg/L L-serine with fetal bovine serum (FBS 10%), both at 37°C, and 50 *μ*L cell suspension, for a final cell density of 2 × 10^5^ cells/mL. This suspension was maintained in MEM Eagle 1.8 mMCa^++^ containing FBS (10%), streptomycin (100 mg/L), and penicillin (70 mg/L). Then, the plates were placed in an incubator with an atmosphere of 5% CO_2_ at 37°C for 24 hours, for adhesion of macrophages. For cell treatment, the equivalent of 10% of the total volume (100 *μ*L) of negative (DMSO final concentration = 1%) or positive controls or Infertile at 6, 12, 30, and 60 mM was added, and the plates were incubated (atmosphere of 5% CO_2_ at 37°C) for 3 hours. After this period, the medium was removed and the plates were washed with 1 mL MEM Eagle 1.8 mM Ca^++^. 1 mL Eagle MEM medium 1.8 mM Ca^++^ with FBS (10%) was added and the medium was incubated for 24 hours in an atmosphere of 5% CO_2_. The negative control used in the assay was 100 *μ*L DMSO, while the positive control was 100 *μ*L N-methyl-N-nitro-N-nitrosoguanidine (MNNG) at a concentration of 0.5 mM. To determine the mitotic index and the number of micronuclei, the MEM Eagle 1.8 mM Ca^++^ solution was replaced with cold Carnoy's fixative (3 : 1 methanol to glacial acetic acid) for 15 minutes. The coverslips were washed with McIlvaine's buffer (MIB) (21.01 g/L citric acid and 35.60 g/L Na_2_HPO_4_, pH 7.5) for 2 minutes and left to dry at room temperature. The cells were then stained with 4′-6-diamidino-2-phenylindole (DAPI) (0.2 *μ*g/mL) dissolved in MIB for 40 minutes, washed with MIB for 2 minutes, and briefly rinsed with distilled water. To determine the mitotic index, the number of cells with micronuclei and the percentages of necrosis and apoptosis, 1000 cells per concentration, were analyzed under a fluorescence microscope (Reichert Univar) with an excitation wavelength of 350 nm. Cells that glowed brightly and had homogenous nuclei were considered as having normal phenotypic morphology. Apoptotic nuclei were identified by the condensed chromatin at the periphery of the nuclear membrane or by fragmented nuclear body morphology. Necrotic cells presented chromatin forms with irregularly shaped aggregates, a pyknotic nucleus (shrunken and darkly stained), and cell membrane disruption, with cellular debris spilled into the extracellular milieu. The experiment was conducted in triplicate [24, 27, 28].

#### 2.2.5. Statistical Analysis

The one-way ANOVA, followed by Tukey's posttest was performed using GraphPad Prism 5.0 for bacterial and eukaryotic cell models. For* Salmonella*/Microssome assay, we also performed Bernstein's correlative analysis using SALANAL software.

## 3. Results and Discussion

The pharmacokinetic properties of Infertile are presented in Table 1. According to pkCSM in silico prediction, zinc gluconate is poorly absorbed and consequently presents low distribution volume and is chemically inert to CYP isoenzymes.

The predictive results presented by pkCSM and LAZAR algorithms were compared and presented in Table 2. Both predictive strategies pointed Infertile as nonmutagenic in Ames toxicity test. LAZAR prediction suggested carcinogenic effect of zinc gluconate in rodents in general and to mice and rats separately. The maximum tolerated dose in humans and the toxicity to fathead minnows were predicted in the same range using both strategies.

These pharmacokinetic aspects of the compound favor its low hepatotoxic profile, once the prediction suggests no interaction between zinc gluconate and CYP enzymes, both used as substrate and inhibitor [29]. According to LAZAR's prediction, the carcinogenicity propensity of Infertile was determinant to the following investigation of the genetic toxicological profile of this compound.

**Figure 2:**
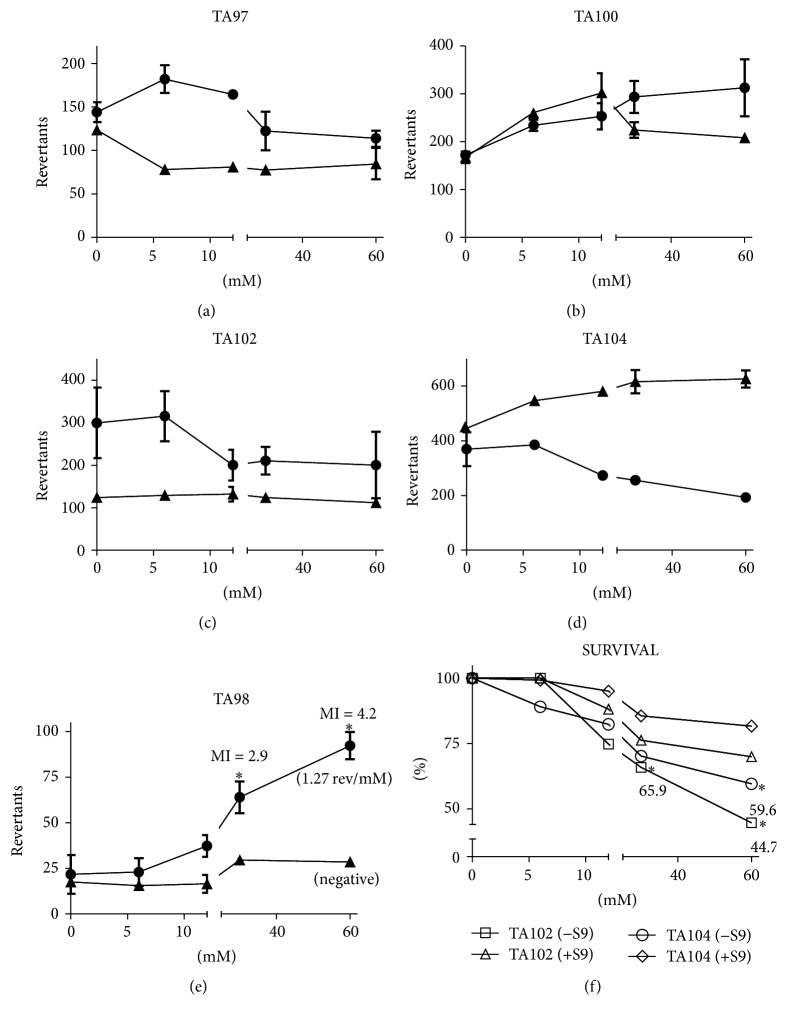
Mutagenicity and cytotoxicity evaluation of Infertile. The graphs ((a), (b), (c), and (d)) show that, in both absence (−S9, ●) and presence (+S9, ▲) of exogenous metabolism, there were no mutagenic concentrations to TA97, TA100, TA102, and TA104. On the other hand, in (e), mutagenic activity to TA98 (−S9) was detected. No cytotoxic (survival ≤ 70%) response was observed to TA97, TA98, and TA100 (−S9/+S9, data not shown). In (f), there was a decrease of survival to TA102 and TA104 at 60 mM (^*∗*^ < 0.01 versus negative control; *n* = 3 in triplicate; one-way ANOVA followed by Tukey's post hoc test).

Although the predictive results using the in silico approach indicated the absence of mutagenicity in Ames test, we performed the bacterial reversion assay (Ames test) using* Salmonella* strains and observed a mutagenic response for Infertile (Figure 2). In the absence of metabolic activation (−S9), TA98 strain indicated a positive mutagenic response (MI ≥ 2, Mutagenic Slope; 1,27 revertants/mM to Infertile at 30 mM and 60 mM; *P* < 0.01). A cytotoxic effect was observed for TA104 (60% survival) at the 60 mM concentration. With metabolic activation (+S9), no mutagenicity was detected for the strains used. However, cytotoxicity was detected for TA102 (65% survival at 30 mM and 45% survival at 60 mM) in the presence of the compound. Infertile showed negative mutagenic responses for strains TA97, TA100, TA102, and TA104 (−S9/+S9). Moreover, no cytotoxic effect was observed for TA97a, TA98, and TA100 strains in the presence or absence of exogenous metabolism in all tested concentrations.

Infertile demonstrated mutagenic potential for the strain TA98 in the absence of exogenous metabolism system, which suggests frameshift mutation by G:C pair deletion. Infertile will probably not represent a risk for animal health because the Sertoli cells in animal testis exhibit a reasonable metabolic capacity to convert the compound into nontoxic metabolites [30]. Besides, the presence of the Zn^2+^ cation in the chemical structure of Infertile has a central role in the toxicity of the molecule, because heavy metals such as zinc, lead, cadmium, and mercury can increase the mutagenicity and cytotoxicity of various compounds in bacterial reverse mutation evaluations. It is likely that, in the +S9 experiments, no mutagenic concentration was observed because of the presence of metalloproteins in the liver homogenate, which bind to metallic cations and reduce their availability [31].

There is evidence that Infertile is a clastogenic or aneugenic compound, because, at 12 mM, almost 10% of the cells were found with micronucleus. At 30 and 60 mM, the increased rate of necrosis and apoptosis suggests high cytotoxicity. The increased rate of apoptosis at 30 mM suggests that silent cell death occurs at this concentration, whereas, at 60 mM, there was an increase in necrosis, pointing out to abrupt cell death, which masks genotoxic response. This fact is crucial for the sterilant activity of Infertile, once the death of the epithelial and Sertoli cells in the seminiferous tubules is necessary for castration to occur [6]. The damage caused by the compound leads to a delay in cellular cycle, which could be the reason of the alteration in the mitotic index.

**Figure 3:**
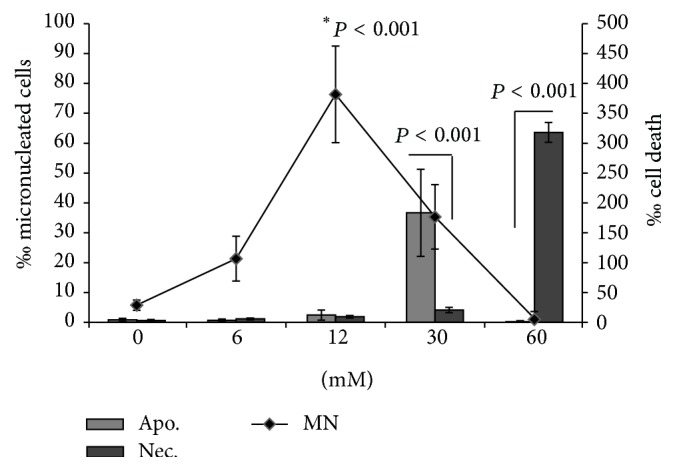
Infertile genotoxicity and cytotoxicity evaluation using RAW267.4 macrophage cell lineage. There are three curves showing the percentage of micronucleated cells (MN) in apoptosis (Apo) and necrosis (Nec). It can be observed that, at 12 mM, there is predominance of micronucleated cells (85/1000 cells), at 30 mM, cells in apoptosis predominate (143/1000 cells) and, at 60 mM of Infertile, there were 318 cells in necrosis/1000. It is important to note that when an event prevails, others tend to diminish. It is possible to observe that there is a turnover among the cell death events (^*∗*^ < 0.01 versus negative control; *n* = 3 in triplicate; one-way ANOVA followed by Tukey's post hoc test).

The results of the macrophage micronucleus assay are shown in Figure 3. At 12 mM, there was an increase in micronucleated cells rate (8%). In higher concentrations (30 and 60 mM), a prevalence of apoptotic and necrotic cells (cell death) was observed.

Infertile is registered by the Brazilian Ministry of Agriculture, Livestock and Supply and the usage dose ranges from 200 mM to 1000 mM [32], which indicates that it underwent a strict quality control before it could be in the market. So far, chemical castration methods, as the intratesticular injection of Infertile, have not promoted any major harm to animals; therefore, they have the approval and support of specific organs, such as the Alliance for Contraception in Cats & Dogs and the Regional Council of Veterinary Medicine of the State of São Paulo [33]. However, either the product or the method still needs to be improved.

A study in Galapagos Islands showed that some animals treated with the zinc gluconate procedure presented tissue necrosis. These complications were attributed to improper injection techniques or inaccurate after-treatment management, besides the intrinsic characteristics of the local environment [34]. The evidence of tissue necrosis was linked to external events whereas it could have been related to an endogenous reaction based on inflammatory response. The presence of inflammation after the intratesticular injection of 200 mM zinc gluconate was reported, and anti-inflammatory drugs were prescribed to minimize this side effect [35].

The detection of cytotoxic concentrations of Infertile on eukaryotic cells in the present study corroborates previous findings for in vivo experiments [34, 35].

When cell death is silent, as in apoptosis, there is no inflammatory response, and dying cells contract into an almost invisible corpse that is soon consumed by neighboring cells. On the other hand, necrosis releases proinflammatory signals to the surrounding tissue microenvironment, unlike apoptosis [36]. As a consequence, necrotic cells induce an immune response to recruit inflammatory cells. Moreover, necrotic cells can release chemokines and bioactive factors that can stimulate viable cells to proliferate, with the potential, once again, to facilitate neoplastic progression [37].

Necrosis has a crucial role in inflammatory response. Once cellular disruption occurs, several damage associated molecular patterns (DAMPs) are released, such as mitochondrial DNA (mtDNA), which exerts immunogenic function and can recruit neutrophils to the area of necrosis [38]. The presence of DAMPs originated from cell debris at necrotic sites (necrotaxis signals) has been described as being more important than chemotactic stimuli for establishing leukocyte migration and inflammatory response [39]. A chronic necrotic-induced inflammatory environment causes the emergence of DNA damage induced by oxidative stress. This phenomenon can be mediated by ROS, such as superoxide radicals (O_2_^∙−^) and RNS, derived from nitric oxide radicals (NO^*∙*^).

Oxidative damage can lead to single- or double-strand breaks, frameshift and point mutations, and chromosome abnormalities, and more than 30 different products of DNA and RNA nucleobases produced by oxidative damage have been identified [40]. Besides, TNF-*α*, an inflammatory cytokine, can trigger inflammation-mediated carcinogenesis. The molecular basis possibly involves induction of reactive oxygen. Reactive oxygen in the form of NO is often generated by inflammatory cytokine induction of NO synthase. NO can directly oxidize DNA, resulting in mutagenic changes, and it may cause damage to some DNA repair proteins. Inflammatory cytokines may also affect genome integrity via inhibition of cytochrome P450 or glutathione S-transferase isoenzymes [41].

Recently, it was demonstrated that nanoparticles of zinc oxide can delay apoptosis, reinforcing an oxidative cellular microenvironment and an increase in proinflammatory response, by enhancing the secretion of IL-1*β* and IL-8 in human cells [42]. The combination of these three factors suggests that zinc can favor a procarcinogenic environment in exposed tissues.

## 4. Conclusions

The cytotoxic effect of the zinc gluconate-based product, Infertile, is an important factor, considering its use as a canine sterilization agent. Cell death mechanism would have to be apoptosis, since necrotic processes are potentially carcinogenic. Although Infertile is licensed for use on animals, its genotoxic and cytotoxic effects, shown in the in vitro toxicological evaluation, demonstrate that the highest dose (60 mM) presents a risk for animal health by necrosis induction. Studies must be continued in order to clarify the activity on cells and tissues involved in the sterilant activity of Infertile and the cell damage induced, in order to better understand the pathophysiological mechanisms of this drug.

## Figures and Tables

**Table 1:** Infertile's pharmacokinetic (ADME) properties prediction in pkCSM analysis of similarity.

Property	Model name	Predicted value	Unit
Absorption	Water solubility	2.99	Numeric (mmol/L)
Absorption	Caco2 permeability	−0,8980	Numeric (log Papp in 10^−6^ cm/s)
Absorption	Intestinal absorption (human)	0,0000	Numeric (% Absorbed)
Absorption	Skin permeability	−2.7350	Numeric (log Kp)
Absorption	P-glycoprotein substrate	Yes	Categorical (yes/no)
Absorption	P-glycoprotein I inhibitor	No	Categorical (yes/no)
Absorption	P-glycoprotein II inhibitor	No	Categorical (yes/no)
Distribution	VDss (human)	44.9	Numeric (mL/kg)
Distribution	Fraction unbound (human)	0.8810	Numeric (Fu)
Distribution	BBB permeability	−2.2160	Numeric (log BB)
Distribution	CNS permeability	−6.1620	Numeric (log PS)
Metabolism	CYP2D6 substrate	No	Categorical (yes/no)
Metabolism	CYP3A4 substrate	No	Categorical (yes/no)
Metabolism	CYP1A2 inhibitor	No	Categorical (yes/no)
Metabolism	CYP2C19 inhibitor	No	Categorical (yes/no)
Metabolism	CYP2C9 inhibitor	No	Categorical (yes/no)
Metabolism	CYP2D6 inhibitor	No	Categorical (yes/no)
Metabolism	CYP3A4 inhibitor	No	Categorical (yes/no)
Excretion	Total clearance	4.06	Numeric (ml/min/kg)
Excretion	Renal OCT2 substrate	No	Categorical (yes/no)

VDss: volume of distribution at steady state; BBB: brain blood barrier; CNS: central nervous center; CYP: cytochrome P; OCT: organic cation transporter.

**Table 2:** Comparison between pKCSM and LAZAR toxicity prediction of Infertile.

Model Name	pkCSM		LAZAR	
	Unit	Prediction	Unit	Prediction
AMES toxicity	Categorical (yes/no)	No	Categorical (yes/no)	No
Hepatotoxicity	Categorical (yes/no)	No	n.a.	—
Skin sensitisation	Categorical (yes/no)	No	n.a.	—
hERG I inhibitor	Categorical (yes/no)	No	n.a.	—
hERG II inhibitor	Categorical (yes/no)	No	n.a.	—
Oral rat acute toxicity (LD50)	Numeric (mol/kg)	0.29	n.a.	—
Oral rat chronic toxicity (LOAEL)	Numeric (mg/kg/day)	22.49	n.a.	—
Carcinogenicity (rat)	n.a.	—	Categorical (Yes/No)	Yes
Carcinogenicity (mouse)	n.a.	—	Categorical (Yes/No)	Yes
Carcinogenicity (rodents)	n.a.	—	Categorical (Yes/No)	Yes
Max. tolerated dose (human)	Numeric (mg/kg/day)	16.11	Numeric (mg/kg/day)	7.93
*T. pyriformis* toxicity	Numeric (log ug/L)	1.93	n.a.	—
Fathead minnow toxicity	Numeric (mol/L)	1,164	Numeric (mol/L)	1,567

hERG: human Ether-à-go-go-Related Gene; LD50: lethal dose of 50%; LOAEL: lowest observed adverse effect level; n.a.: not analyzed.
